# Successful pregnancies in post-kidney transplant couples: four case reports

**DOI:** 10.3389/fimmu.2023.1215480

**Published:** 2023-07-12

**Authors:** Hao Huang, Xinyu Liu, Xiaoli Lin, Xiaoying Wu, Yingyin Qiu, Hongfeng Huang

**Affiliations:** ^1^ Department of Rehabilitation Medicine, Renji College of Wenzhou Medical University, Wenzhou, China; ^2^ Kidney Disease Center, The First Affiliated Hospital, Zhejiang University School of Medicine, Hangzhou, China; ^3^ Key Laboratory of Nephropathy, Hangzhou, Zhejiang, China; ^4^ Institute of Nephropathy, Zhejiang University, Hangzhou, China

**Keywords:** kidney transplantation, couple, pregnancy, immunity, pre-eclampsia

## Abstract

**Background:**

The fertility of female kidney transplant recipients is increasing with the progression of transplant management. This article aims to evaluate the clinical prognosis of mothers and newborns for post-kidney transplant couples.

**Methods:**

From January 2019 to April 2022, a total of four couples, all kidney transplant recipients, were successfully prepared for pregnancy after a rigorous preconception evaluation, including three cases of natural conception and one case of *in vitro* fertilization. Data regarding the mother and newborn, including general clinical condition and laboratory results, were recorded and assessed throughout the pregnancy and up until 12 months after delivery.

**Results:**

The mean conception age of the mothers was 34.8 years (30–38 years), and the mean interval between renal transplantation and pregnancy was 6.6 years (3.7–8.7 years). All deliveries were by cesarean section and took place without incident. There were three premature births (<37 weeks; average 35.1 weeks). In case 1 (*in vitro* fertilization), pre-eclampsia occurred during maternity, and this was the only case in which the fetal weight was less than 2,500 g (average 2,576.7 g). The mean Apgar score (1 min) was 7.8 (6–9) and reached 9 in all cases at 5 min. The mothers’ eGFR rose during mid-gestation, decreased in late pregnancy, and was largely restored along with proteinuria 1 year postpartum. Postnatal evaluation at 6 months showed normal neurological development. In addition, NK cell and IFN-γ levels increased and Treg cell and IL-10 levels decreased along with the onset of pre-eclampsia.

**Conclusions:**

Pregnancies can succeed in couples who are both kidney transplant recipients. However, there might be higher risks of infertility, prematurity, and low birth weight.

## 1 Introduction

Kidney transplantation has become the ideal alternative treatment for patients with end-stage renal disease due to continued improvements in the transplantation process and subsequent patient management (1). Along with further stabilization of renal function and adequacy to meet metabolic demands, most female recipients of childbearing age experience a marked improvement in quality of life and substantially restored reproductive function. Successful pregnancy in female kidney transplant recipients was first reported in 1963 (2).

As the number of kidney transplant recipients continues to increase, the rate of pregnancy in this group grows accordingly each year due to ideal transplant kidney function and the pursuit of a higher quality of life (3, 4). It has been reported that, by 2011, more than 11,000 women world wide have been at childbearing age after kidney transplantation (5). In 2018, the International Pregnancy Registry (TPR) reported 1,993 pregnancies in 1,101 kidney transplant recipients in the US. Although the current living birth rate reaches 72% to 80%, compared with the general pregnancy population, pregnancies after kidney transplants were still associated with a significantly increased risk of adverse fetal and maternal pregnancy outcomes (6). Those higher risks are associated with higher incidences of cesarean delivery (56.9% versus 31.9%) and preterm delivery (<37 weeks of gestation), as well as low birth weight (mean birth weight of 2,420 g versus 3,298 g). Additionally, there is an increased risk of complications, such as maternal hypertension and proteinuria, during pregnancy and the development of preeclampsia; while the incidence of acute rejection was relatively low (4.2%) (7).

There has only been one reported case of a successful pregnancy in which both the husband and wife were solid organ transplant recipients. The wife was a liver transplant recipient, and the husband was a kidney transplant recipient, and the twin pregnancy was successfully achieved using the artificial insemination-embryo transfer technique. A cesarean section was performed at 34 weeks of gestation due to pre-eclampsia and premature rupture of membranes (8). It is possible that couples in which both partners are kidney transplant recipients may exist and have a desire to have children. However, whether pregnancy in kidney transplant couples is safe and how it should be managed still lacks consensus.

There have now been more than 130 post-kidney transplant pregnancies in our center, which is the largest amount in a single center in China. In the present study, we report a small case series of four successful pregnancies in post-kidney transplant couples.

## 2 Methods

### 2.1 Study population

From January 2019 to May 2022, four patients, in which both the husband and wife were renal transplant recipients, underwent postoperative pregnancy and delivery in the First Affiliated Hospital, College of Medicine, Zhejiang University, including three cases of natural conception and one case of artificial *in vitro* conception. The immunosuppression regimen was tacrolimus + mycophenolate mofetil + prednisone in all 8 patients. The intervals between surgery and pregnancy were 3.7, 8.7, 6.9, and 7.8 years (average 6.78 years) for the husbands, and 3.7, 8.7, 6.9, and 7.2 years (average 6.63 years) for the wives, respectively. The immunosuppressive drugs for the husbands were not adjusted before pregnancy. Adjustments for wives began 3 months before pregnancy preparation: mycophenolate mofetil was switched to azathioprine 1 mg/kg.d^-1^, prednisone was maintained at 10mg/d, and tacrolimus blood concentrations were controlled at 5–6.5 ng/ml. Thiopurine methyltransferase (*TPMT*) and nucleoside diphosphate-linked moiety X type motif 15 (*NUDT15*) gene polymorphism testing of the azathioprine drug-metabolizing enzyme was required before the drug regimen change.

### 2.2 Data collection and evaluation

#### 2.2.1 Data collection

All patients were routinely followed up and blood and urine samples were collected to evaluate general conditions, liver function, renal function, urine protein/creatinine ratio (UPCR), lipid glucose status, tacrolimus whole-blood valley concentrations, etc. from the beginning of pregnancy preparation. Clinical follow-up and laboratory tests were performed once a month up until at least 1 year after delivery. During pre-eclampsia or the prenatal stage, the number of NK cells and Treg was detected by flow cytometry, and the levels of cytokines IFN-γ and IL-10 were detected by the clinical laboratory using the ELISA method.

#### 2.2.2 Basic requirements for permitting pregnancy

Wife: 1. An interval between kidney transplantation and pregnancy of >2 years and ideal general health condition; 2. Stable renal function (serum creatinine of <176 μmoI/L, optimally <132 μmoI/L); 3. without transplant rejection within the past 6 months; 4. Strict blood pressure control with or without antihypertensive drugs; 5. No proteinuria (urine protein of <300 mg/d or UPCR of <300 mg/g); 6. Ultrasound reported a normal transplanted kidney, with no manifestation of hydronephrosis or kidney stones; 7. Recommended combination regimen of immunosuppressive drugs before and during pregnancy, as listed above (9, 10).

Husband: 1. Renal function in the normal range or stabilized; 2. Suggested to quit smoking and alcohol in the past 3 months, avoids staying up late, strong tea, and stimulating beverages and food; 3. Without sperm quality-affecting drug use in the past 3 months, including cyclophosphamide, colchicine, sulfonamides, moxifloxacin, and spironolactone diuretics; 4. Recommended immunosuppressive drug regimen: calcineurin inhibitor (tacrolimus or cyclosporine) + mycophenolic acid/azathioprine + prednisone; 5. Confirmed that the spermatic cord was not ligated during the kidney transplant operation, and if necessary, semen tests can be performed earlier (11).

#### 2.2.3 Evaluation of the newborns

The Apgar score system is a newborn health status measuring tool that takes appearance, pulse, grimace, activity, and respiration into account and is applied 1, 5, and 10 min after delivery. The total score is 10 and a score of ≥8 is in the normal range. A score of 4–7 is classified as mild asphyxia and 0–3 is classified as severe asphyxia that requires emergency rescue.

The neurological development of newborns born 6 months postpartum was evaluated using the Neonatal Behavioral Neurological Assessment (NBNA) (12, 13). The NBNA consists of 20 assessment items divided into five sections: behavioral ability (6 items), passive muscle tone (4 items), active muscle tension (4 items), primitive reflex (3 items), and general assessment (3 items). Each score has three grades (0, 1, and 2). The total score is 40 and a score of ≥35 indicates normal development of the nervous system. Higher scores imply better behavioral nerve development.

#### 2.2.4 Diagnostic criteria for pre-eclampsia

Systolic blood pressure of ≥140 mmHg and/or diastolic blood pressure of ≥90 mmHg after 20 weeks of gestation (at least twice, with a measurement interval of at least 4 h); concomitantly with 24-h urine protein of ≥0.3 g or random urine protein/creatinine of ≥300mg/g, or random urine protein count ≥0.3g (14).

### 2.3 Ethical statement

All organ procurement adhered to the guidelines of the National Plan for Deceased Organ Donation in China, the Helsinki Congress, and the Istanbul Declaration. Local organ procurement organizations and Red Cross organizations were responsible for organ procurement and distribution. All organ donations were voluntary, and no organs were obtained from executed prisoners. Recipients were registered in the Chinese Organ Transplant Response System (COTRS) and signed written informed consent for the procedure. The procedures and transplants described above were performed under the supervision of the OPO, the Red Cross Organization, and the Organ Transplantation Ethics Committee. Informed consent was waived because the study relied on past clinical data collection, did not involve any identifying information, and did not cause any harm to the subjects.

## 3 Results

### 3.1 Baseline clinical data

Three of the four couples were natural conception and case 1 had a pregnancy by the *in vitro* fertilization-embryo transfer (IVF-ET) technique due to oligospermia. All newborns were delivered alive by cesarean section, including 3 cases of preterm delivery (<37 weeks). The average gestational time was 35.1 weeks (32.2–38.0 weeks). The mean conception age of the mothers was 34.8 years (30–38 years), and the mean interval between renal transplantation and pregnancy was 6.6 years (3.7–8.7 years) (**Table 1**).

**Table 1:** Baseline clinical data of the four couples.

pair number	role	conception method	primary kidney disease	age at conception	BMI before pregnancy	Interval between surgery and pregnancy (years)	Gestational weeks (week)
1	wife	*in vitro* conception	unavailable	30	20.2	3.7	32.2
	husband		chronic glomerulonephritis	33	20.8	3.7	
2	wife	natural conception	allergic purpura	38	30.8	8.7	36.1
	husband		chronic glomerulonephritis	37	25.3	8.7	
3	wife	natural conception	chronic glomerulonephritis	34	27.6	6.9	38.0
	husband		chronic glomerulonephritis	39	24.7	6.9	
4	wife	natural conception	chronic glomerulonephritis	37	30.8	7.2	34.2
	husband		chronic glomerulonephritis	43	23.0	7.8	
average (wife)				34.8	27.4	6.6	35.1

### 3.2 Maternity assessment

There was one case of pre-eclampsia (case 1, *in vitro* fertilization). At 28 weeks of gestation, the patient developed hypertension (145/95 mmHg) with a 24-h urine protein of 500 mg. Her proteinuria rapidly progressed to 2,600 mg of 24-h urine protein at 32 weeks, and her pregnancy was terminated at 32.2 weeks due to excessive proteinuria and hypertension. The proteinuria gradually recovered postpartum, while her renal function became stable but increased compared with pre-pregnancy (serum creatinine of 97 μmol/L before pregnancy vs. stable serum creatinine of 118 μmol/L 1 year after delivery).

**Figure 1:**
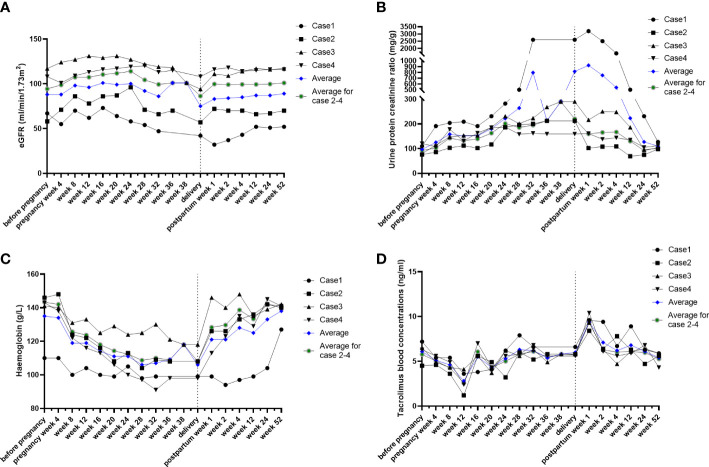
Conventional indicators during post-kidney transplant maternity. The individual course of eGFR **(A)**, urine protein creatinine ratio **(B)**, hemoglobin **(C)**, and tacrolimus blood concentrations **(D)**. Individuals, black lines; average level, blue lines.

The dynamic changes in renal function, urinary protein/creatinine ratio, hemoglobin, and whole-blood trough concentration of tacrolimus in four cases of pregnancy were demonstrated as follows:

Renal function (**Figure 1A**): estimated GFR increased from 8 weeks of gestation and reached a peak at 16 weeks of gestation, which increased for around 20 ml/min/1.73 m2 compared with pre-pregnancy. Their eGFR gradually recovered postpartum except for case 1, whose eGFR decreased for 15 ml/min/1.73 m2 compared with pre-pregnancy.

Urine protein creatinine ratio (UPCR) (**Figure 1B**): despite the obvious increase in case 1, the urine protein creatinine ratio gradually increased since pregnancy week 8 until delivery in the other three cases. UPCR in all patients gradually returned to the normal range postpartum.

Hemoglobin (**Figure 1C**): hemoglobin decreased from 8 weeks of gestation, reached trough values at 16–20 weeks (averagely decreased 2.4 g/L), and gradually recovered postpartum.

Tacrolimus blood concentrations (**Figure 1D**): tacrolimus blood concentrations decreased from 8 weeks of gestation and were maintained at 5–6 ng/ml after adjusting the drug dosage to approximately 30%. The concentrations dramatically increased by nearly twofold and returned to a sustained level of 5–6 ng/ml after prompt drug adjustment.

### 3.3 Immune cell and cytokine analysis in the prenatal period

**Figure 2:**
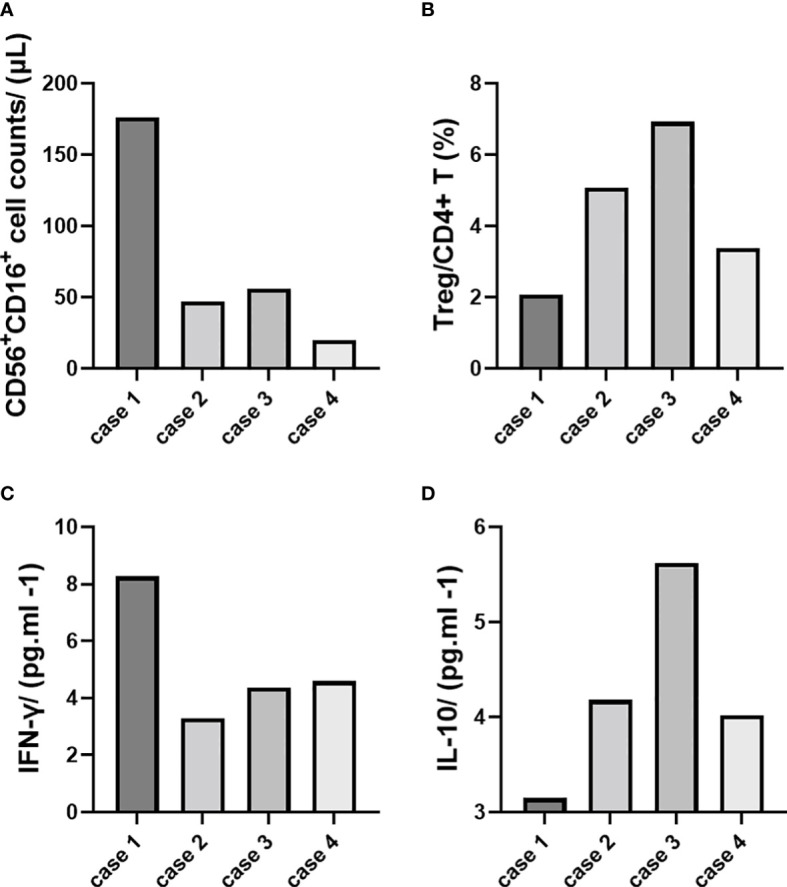
Immune cell and cytokine analysis (demonstrated with single cases). **(A)** CD56+CD16^+^ cell counts/(μl). **(B)** Treg/CD4^+^ T (%). **(C)** IFN-γ/(pg.ml ^-1^). **(D)** IL-10/(pg.ml ^-1^).

We conducted immune cell and cytokine analysis to explore the innate immunity experience during the prenatal period. An obvious increase in NK cell counts and IFN-γ was found in case 1 (accompanied by pre-eclampsia), while Treg cell and IL-10 levels were lower than in other cases (**Table 2**; **Figure 2**).

**Table 2:** Comparison of NK, Treg cells and related cytokines in the peripheral blood.

pair number	CD56 ^+^ CD16 ^+^/NK (/μL)	Treg/CD4^+^ T (%)	IFN-γ/ (pg.ml ^-1^)	IL-10/ (pg.ml ^-1^)
1	176	2.07	8.27	3.15
2	47	5.07	3.29	4.18
3	56	6.92	4.37	5.62
4	20	3.38	4.59	4.02

### 3.4 Neonatal assessment

All newborns were alive at birth. The average birth weight was 2,576.7 g (2,250–3,250g), with one case less than 2,500g (case 1, *in vitro* fertilization). Their Apgar score was 7.8 at 1 min (lowest at case 1) and over 9 at both 5 and 10 min. Neonatal Behavioral Neurological Assessment (NBNA) scores were all in the normal range 6 months after birth (**Table 3**).

**Table 3:** Neurological development evaluation of the newborns.

pair number	Gestational weeks (week)	Birth weight (g)	Apagar score (1 min)	Apagar score (5 min)	Apagar score (10 min)	NBNA score (6 months postpartum)
1	32.2	2150	6	9	10	36
2	36.1	2540	7	9	10	37
3	38.0	2440	9	10	10	38
4	34.2	2750	9	10	10	37
average	35.1	2577	7.8	9.5	10	37

NBNA, Neonatal Behavioral Neurological Assessment.

## 4 Discussion

After patients with end-stage renal disease successfully receive kidney transplantation, their fertility is typically restored approximately 6 months after surgery as renal function and hypothalamic-pituitary-ovarian axis function gradually return to normal. Data from OPTN/SRTR show that approximately one-third of kidney transplant recipients in 2019 were 18 to 49 years old at the time of transplantation, with 28–40% of them being female. Therefore, female recipients of childbearing age might account for 15% of all the recipients, even without accounting for female adolescent recipients who will grow to adulthood (15).

There have been more than 130 post-kidney transplant pregnancies in our center, among which there are 4 special cases in which the husbands were also post-kidney transplant recipients with stable renal function, and the couples received their surgery almost at the same time and in the same hospital. These four recipients were strictly evaluated by multiple departments, including renal transplantation, nephrology, and obstetrics departments before conception. Three of them conceived spontaneously and one conceived by *in vitro* fertilization due to oligospermia of the husband. After close follow-up and management during pregnancy, one patient delivered at 32.2 weeks of gestation due to pre-eclampsia (hypertension and proteinuria of 2.6 g/d at 32 weeks of gestation). The others delivered successfully at 34.2, 36.1, and 38 weeks of gestation. All of them were followed up for more than 1 year, showing stable renal function, no proteinuria, and neurological NBNA scores of the newborn babies in the normal range 6 months after birth.

The specificity of pregnancy in couples who are both kidney transplant recipients concentrates on the preconception period. Assessments of female kidney transplant recipients in this study mainly referred to the recommended guidelines of the European Society of Dialysis and Transplantation published in 2002 and the guidelines of the American Society of Transplantation published in 2005 (9, 10). Three of them were spontaneously conceived, and one was conceived through *in vitro* fertilization due to her husband’s oligospermia. In addition to the routine assessment of the wife’s pregnancy eligibility, it is also important to pay attention to the husband who is also a kidney transplant recipient. Ligation or compression of the spermatic cord during kidney transplantation in male recipients, long-term use of immunosuppressive agents, and poor physical condition can lead to infertility. Case 1 had not been able to conceive successfully for 3 months during and confirmed that his spermatic cord was not ligated during operation. Semen examination showed that he had oligospermia. After *in vitro* fertilization and embryo transfer, successful conception was achieved. According to previous studies, male kidney transplant recipients who use calcineurin inhibitor (tacrolimus or cyclosporine), mycophenolic acid, and prednisone before pregnancy usually do not need to change their immunosuppressive regimen (16).

Owing to the clear advantages of azathioprine, such as a lower spontaneous abortion rate, teratogenicity, and good safety of breastfeeding, it is generally recommended as an anti-metabolic drug during pregnancy in female recipients (17). It is necessary to convert mycophenolic acid to azathioprine 3 months before pregnancy preparation. However, some patients may be intolerant to azathioprine and may experience severe adverse drug reactions, such as bone marrow suppression and severe liver function damage. If the medication is not stopped promptly, it can even lead to death (18). Understanding of azathioprine drug-related toxicities was previously limited to the mercaptopurine methyltransferase (*TPMT*) gene polymorphism, but recently it has been found that *TPMT* gene polymorphism cannot fully explain the associated severe myelotoxicity in Asian populations. Studies (19, 20) have shown that *NUDT15* gene variants can cause azathioprine-associated leukopenia, and the mutation rate of this gene can be up to 15% in Asian populations, which is significantly higher than in Westerners. Therefore, renal transplant recipients who switch to azathioprine need to undergo both *TPMT* and *NUDT15* gene polymorphism testing to reduce azathioprine-related toxicity. Fortunately, preconception genetic screening in all four female recipients in this study did not reveal any abnormalities.

Pregnancy after renal transplantation has become increasingly common. However, the risks of pregnancy-related complications still increased compared with pregnancies without transplantation background and conceived spontaneously, which respond to gestational hypertension, pre-eclampsia (23.6–38.1%), proteinuria, and decreased postpartum renal function (21, 22).

Pre-eclampsia, in particular, requires attention. The dynamic balance between pro-inflammatory and anti-inflammatory mediated by immune cells and cytokines during pregnancy maintains the stability of the microenvironment at the fetal-maternal interface, and once the balance is disturbed, it can lead to pregnancy complications (23, 24). Non-invasive monitoring indicators are currently the research focus, and changes in NK and Treg cell counts in peripheral blood may indirectly reflect the inflammatory or tolerogenic status of the placenta (25, 26). Therefore, monitoring peripheral blood NK and Treg cells and the corresponding cytokines IFN-γ and IL-10 is of clinical importance in determining disease prognosis and optimizing treatment strategies, providing the possibility of the early diagnosis of pre-eclampsia (27). In this study, we demonstrated that the level of CD56+CDl6+ NK cells and IFN-γ increased in case 1 with pre-eclampsia, while Treg and IL-10 decreased.

Additionally, there were higher incidences of preterm delivery (43.1%–48.5%) and low birth weight babies (43.7%–56.3%). Furthermore, it was found that the stability and fluctuation of renal function in the year before pregnancy and renal hyperfiltration during pregnancy were significant risk factors for adverse events during pregnancy and the deterioration of renal function after delivery (6, 28–30). In this study, three of the four recipients delivered prematurely, with a mean gestation time of 35.1 weeks and a mean neonatal weight of 2,577 g. This small sample study suggested that pregnancy outcomes were essentially similar between post-kidney transplant couples and single post-kidney transplant mothers.

The uniqueness of this study is that the husbands were also kidney transplant recipients. First, long-term immunosuppressive drug usage may affect the quantity and quality of sperm. Second, the spermatic cord potentially affected by ligation or compression of local adhesions during kidney transplantation can cause infertility (31). Data from the Norwegian Transplant Registry and the Norwegian Medical Birth Registry for the period 1967–2009 showed that all 2,463 Norwegian men who underwent solid organ transplantation were fathers of 4,614 deliveries before and 474 deliveries after transplantation. There was an increased risk of pre-eclampsia after transplantation compared with pre-transplantation (AOR: 7.4, 95% CI: 1.1–51.4). No increased risk of congenital malformations or other adverse outcomes was found compared with pre-transplant pregnancies or the general population (2,511,506 deliveries) (11), which may reassure male transplant recipients who are planning to have children.

However, it should be noted that the chances of infertility may still increase in couples who are both renal transplant recipients. In this study, one recipient was successfully conceived by artificial insemination-embryo transfer due to the husband’s oligospermia (case 1). The gestation period was only 32.2 weeks and the neonatal weight was the lightest at 2,150 g, consistent with the findings of some previous studies that the use of *in vitro* fertilization method can lead to an increase in preterm delivery and low birth weight babies (32–34). Thus, *in vitro* fertilization may lead to more fetal risks than natural conception (35, 36). For late adverse outcomes, the neurological development of newborns is also a major concern. The NBNA scoring method is generally used to reflect brain development in preterm infants and its prognostic value is better than cranial ultrasound and CT. Reported sensitivity and specificity of the NBNA for brain development on postnatal day 7 were 88.9% and 82.6%, and for brain development on postnatal days 12–14 were 84.6% and 97.4%, respectively (37). In this study, the NBNA scores were obtained for newborns at 6 months and all scored greater than 35; no other developmental abnormalities were found during follow-up.

In conclusion, the present study is a small sample size clinical case report in a single center reporting pregnancies in post-kidney transplant couples with a strict evaluation process from preconception to postpartum, as well as in the newborns. However, the chances of infertility, preterm delivery, and low birth weight fetuses may be higher. Additionally, we need to pay attention to the dynamics of postpartum renal function in the mothers, as well as to the development of the newborns in the long term. The limitations of this study are obvious: the small sample size and the inability to verify the reliability of anti-hypertensive drug history due to the absence of laboratory data and its retrospective analysis design. Further conclusions still need to be verified by successive accumulated cases.

## Data availability statement

The raw data supporting the conclusions of this article will be made available by the authors, without undue reservation.

## Ethics statement

The studies involving human participants were reviewed and approved by the Ethics Committee of the First Affiliated Hospital, Zhejiang University School of Medicine. Written informed consent for participation was not required for this study in accordance with the national legislation and the institutional requirements. Written informed consent was not obtained from the individual(s) for the publication of any potentially identifiable images or data included in this article.

## Author contributions

HH, XLiu, and XLin writing and revising the manuscript. XW and YQ: collecting clinical data and statistical analysis. HFH: funding acquisition, reviewing the manuscript. All authors agree to be accountable for the content of the work. All authors contributed to the article and approved the submitted version.
